# Acupuncture Compared with Intramuscular Injection of Neostigmine for Postpartum Urinary Retention: A Systematic Review and Meta-Analysis of Randomized Controlled Trials

**DOI:** 10.1155/2018/2072091

**Published:** 2018-05-14

**Authors:** Xiao-Mei Wang, Jing Gong, Si-Cong Li, Mei Han

**Affiliations:** ^1^Beijing Obstetrics and Gynecology Hospital, Capital Medical University, Beijing, China; ^2^Beijing Anzhen Hospital, Capital Medical University, Beijing, China; ^3^Beijing University of Chinese Medicine, Beijing, China

## Abstract

**Objective:**

To compare the effectiveness and safety of acupuncture versus intramuscular injection of neostigmine.

**Methods:**

Databases including CNKI, VIP, WanFang, SinoMed, PubMed, Cochrane Library, and clinicaltrials.gov database were retrieved for relevant literature, with the retrieval deadline being November 2017. Two reviewers independently screened, selected, and extracted data and validated the results. The methodological quality was evaluated with the “Risk of Bias” tool, and the meta-analysis was performed by using the RevMan 5.3.5 software.

**Results:**

Totally 953 patients with postpartum urinary retention from 15 randomized controlled trials entered the meta-analysis. 12 articles compared the clinical cure rate of acupuncture alone versus intramuscular injection of neostigmine and found the cure rate in the acupuncture group was 2 times that in the neostigmine group (RR, 1.91; 95% CI: 1.66–2.19). 15 articles compared the clinical effectiveness rate of acupuncture alone with that of intramuscular injection of neostigmine and found the clinical effectiveness rate was 28% higher in the acupuncture group than in the neostigmine group (RR: 1.28; 95% CI: 1.16–1.42). No adverse event was reported in the acupuncture group.

**Conclusion:**

Acupuncture alone is more effective in treating postpartum urinary retention than intramuscular injection of neostigmine, with good safety profile. Therefore, it is a feasible and valuable technique in clinical settings.

## 1. Introduction

Postpartum urine retention (PUR) refers to the inability to pass urine for 6 to 8 hours after delivery. Clinically it is manifested as weak dribble of urine or completely blocked urinary tract, accompanied by abdominal distention and pain (symptomatic PUR) [1]; or patients are able to void spontaneously but have a postvoid residual bladder volume (PVRV) of ≥150 mL (symptomatic PUR) [2]. Reported prevalence for overt symptomatic PUR ranges from 0.3% to 4.7% [3, 4]. For asymptomatic PUR, prevalence of even up to 45% has been reported [5]. It can increase postpartum hemorrhage and is not conducive to milk secretion [6].

Postpartum urinary retention is initially treated with routine methods for inducing urination induction. When these routine methods fail, medications (neostigmine, phenoxybenzamine, and enema), acupuncture, and/or catheterization may be applied. Although catheterization is the most effective way to relieve urinary retention, it can increase the risk of urinary tract infection [7]. Intramuscular injection of neostigmine is only effective in 70% of patients, and it is associated with many side effects such as bradycardia, bronchoconstriction, increased secretions, nausea, and vomiting [8]. Therefore, more safe and effective treatments need to be explored and applied.

Acupuncture, as a nondrug therapy, has been widely used in the treatment of various diseases both in China and abroad, especially for pain symptoms and nerve dysfunction. Urination of the bladder is a reflex activity of nerves. Modern medical studies have proved that acupoints are quite consistent with the corresponding viscera in the ganglions. For urinary retention patients with intact bladder nerves, acupuncture of acupoints including pangguangshu, zhongji, ciliao, and shenshu can stimulate the conduction of parasympathetic nerve impulses via nerve reflex, resulting in contraction of bladder detrusor, increase of intravesical pressure, and relaxation of internal sphincter; by doing so, it can regulate the urination function of the bladder [9]. As a safe, effective, and efficient therapy, acupuncture has become increasingly popular among patients. Meanwhile, a large number of studies have investigated the role of acupuncture in treating postpartum urinary retention. In our current analysis, we compared the effectiveness and safety of acupuncture versus intramuscular injection of neostigmine, a commonly used drug, in the treatment of urinary retention.

## 2. Methods

### 2.1. Inclusion/Exclusion Criteria

The subjects were mothers with clinically confirmed postpartum urinary retention; the study type was a randomized controlled trial; the interventions included acupuncture (hand acupuncture or electroacupuncture) alone, with unrestricted acupoints or maneuvers; and the control group was treated with intramuscular injection of neostigmine.

### 2.2. Literature Review

Published literature was retrieved in databases including China National Knowledge Infrastructure (CNKI), WanFang Data, SinoMed, CQVIP, PubMed, and Cochrane Library. In the clinicaltrials.gov website we also retrieved the completed but unpublished studies and tracked the results of these studies. Only Chinese and English articles were retrieved, and the deadline was October 2017. The Chinese retrieval words included “postpartum urinary retention”, “acupuncture”, “electroacupuncture”, “scalp acupuncture”, “three-edged needle”, “plum-blossom needle”, “filiform needle”, and “randomization”, and the English retrieval words included “urinary retention”, “acupuncture”, “acusector”, “needle”, “needling”, “randomized controlled trial”, “clinical trial”, “RCT”, “random”, and “randomization”. Based on the characteristics of different databases, retrieval strategies including both subject words + free words and key words + full text were applied.

### 2.3. Literature Screening

The initially identified articles were imported into NoteExpress. Initial screening was performed based on inclusion/exclusion criteria and after reading literature titles and abstracts. Full texts were acquired and read for eligible articles or articles that might meet the inclusion criteria to decide whether they would enter the final analysis.

### 2.4. Data Extraction

A data extraction table was designed to collect the data to be analyzed, which included the following seven aspects: (a) basic information of the enrolled studies; (b) research methods and their possible biases; (c) features of the subjects; (d) interventions; (e) outcome measures; (f) research findings; and (g) other required information. Two reviewers independently extracted these data and validated the findings.

### 2.5. Quality Evaluation

The risk of bias was assessed according to the Cochrane Handbook for Systematic Reviews of Interventions [10], which covered the random sequence generation, allocation concealment, blinding, data integrity, selective reporting of positive and/or negative findings, and other sources of bias. Among them, the “other sources of bias” included the following: (a) were there clear inclusion/exclusion criteria; (b) were the baseline data comparable; and (c) was there any conflict of interest. For selective outcome reporting, since all the included studies did not register their research protocols, the risk of bias was defined as low if the outcome measures included postvoiding residual (PVR) and as high if no PVR was reported. The risk of bias was assessed and validated independently by two reviewers; the results were cross-referenced and any disagreements were dissolved by discussion or consultation of a third evaluator with rich experiences.

### 2.6. Outcome Measures

Primary endpoints are cure rate and PVR; secondary endpoints are overall effectiveness rate; and safety outcome was adverse events.

### 2.7. Data Analysis

The quantitative analysis was carried out by using the Cochrane Collaboration software RevMan5.3.5 for meta-analysis. The relative risk (RR) was selected as the statistic for dichotomous data; the continuous variables are described using mean difference (MD) and 95% confidence interval (CI). During the heterogeneity test, chi-square test was performed firstly; based on its finding, estimates of heterogeneity (*I*^2^) were applied. Fixed-effect model was used when *I*^2^ was ≤50% and the *P* value was ≥0.10; when *I*^2^ was >50% or the *P* value was <0.10, random-effect model was applied. If heterogeneity is high, the source for heterogeneity was explored; subgroup analysis or sensitivity analysis was performed to investigate the stability of meta-analysis.

### 2.8. Measurement of Publication Bias

When a sufficient number of articles (more than 10) were included under the same endpoint addressing the same question (more than 10), a funnel plot was used to measure the publication bias.

## 3. Results

### 3.1. Literature Searching and Screening Flowchart

**Figure 1:**
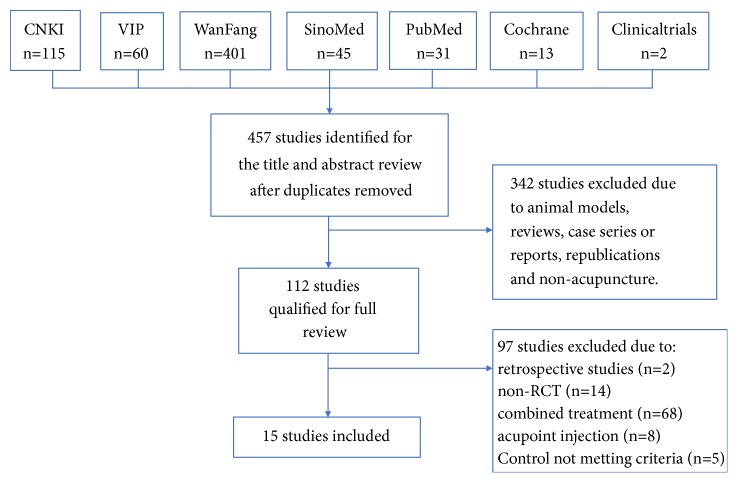
The literature searching and screening flowchart.

Totally 667 articles were retrieved based on our retrieval strategies. After removing duplication and screening the titles and abstracts, we obtained 112 full-text articles. Fifteen articles entered the final analysis according to the exclusion criteria (Figure 1).

### 3.2. Characteristics of Included Literature

A total of 15 randomized controlled trials (RCTs, involving 953 patients) [11–25] on the treatment of postpartum urinary retention by acupuncture alone or intramuscular injection of neostigmine were included in our analysis. The main acupoints used in these studies included sanyinjiao, zhongji, zusanli, guanyuan, yinlingquan, sanjiao, shuidao, and zhiyin. The specific features of these studies are summarized in Table 1.

### 3.3. Quality Evaluation of the Articles

**Figure 2:**
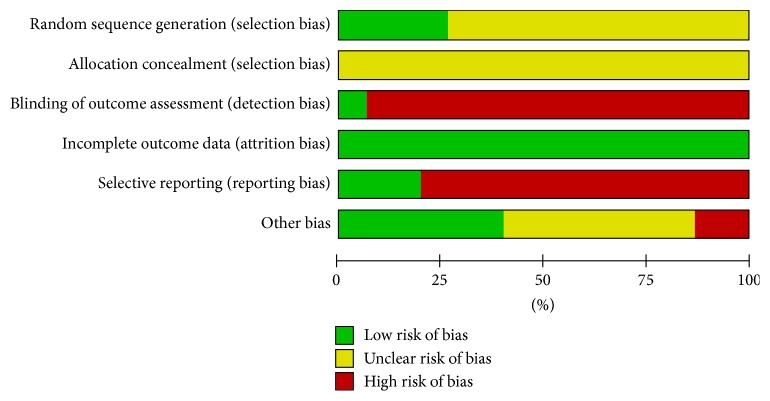
Evaluation of the risk biases of the included studies.

Among these 15 studies, patients were randomized by using random number table in three studies [16, 20, 24] and by using the SPSS software in one study [15]; the remaining 11 studies only mentioned “random” or “randomization,” without describing the specific randomization methods. No literature described allocation concealment. One literature described the blinding of outcome measurers and data analysts [15]. In all literature, the number of patients in all the randomized groups was consistent with the number of subjects in the statistical analysis. Only two articles reported both clinical effectiveness rate and PVR, without selective outcome reporting [15, 21]. The remaining 13 articles only reported the CRR and failed to adequately report RCTs according to the Consolidated Standards of Reporting Trials (CONSORT) statement. The quality of the literature included in our analysis was average. The details of the evaluation are shown in Figure 2.

### 3.4. Results of Meta-Analysis

#### 3.4.1. Cure Rate

**Figure 3:**
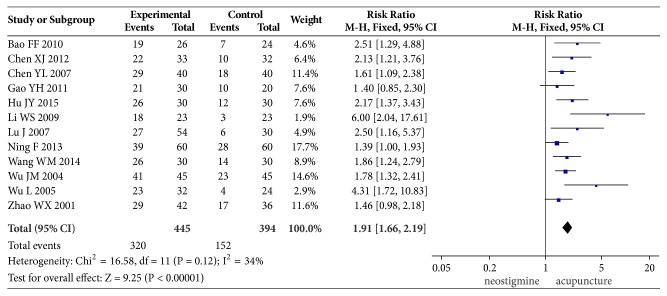
Comparison of the cure rate between acupuncture and neostigmine in the treatment of PUR.

12 of these 15 studies compared the cure rate. While the definition of “cure” differed among these studies, it mainly includes the time of the ability to urinate after treatment (15 min to 3 h was described in 7 trials and insufficient information in the other 4 trials), absence of distending pain in the lower abdomen, and absence of the urgent urination, a PVR of <50 ml, and nonrecurrence. Test for between-studies heterogeneity of 12 studies yielded an *I*^2^ of 34%. The pooled cure rate was 71.91% in the acupuncture group and 38.58% in the neostigmine group; the RR (and 95% CI) was 1.91 (1.66, 2.19) when these two groups were compared in the fixed-effect model, suggesting that acupuncture had a higher cure rate than intramuscular injection of neostigmine in the treatment of postpartum urinary retention. The results of meta-analysis are shown in Figure 3.

#### 3.4.2. Postvoiding Residual (PVR)

Only two articles reported PVR [15, 21], one of which offered means and deviations [21]: the PVR after treatment was 35.45 ± 13.25 ml in the acupuncture group and 70.67 ± 23.58 ml in the neostigmine group (mean: −35.22; 95% CI: −44.90, −25.54), which was even lower in the acupuncture group. In the other article [11], means and interquartile ranges were offered: the median and the interquartile range of PVR after treatment were 0 ml and 1.25 ml, respectively, in the acupuncture group and 89.5 ml and 241.25 ml in the neostigmine group (*P* < 0.001), suggesting acupuncture had a better effectiveness.

#### 3.4.3. Overall Effectiveness Rate

**Figure 4:**
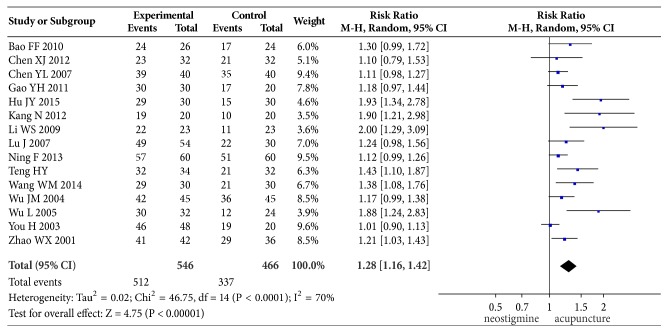
Comparison of the effectiveness rate between acupuncture and neostigmine in the treatment of PUR.

Overall effectiveness rate was reported in all the included 15 articles. The criterion of “effective” differed among different studies, which might have included ability to urinate after treatment but with the presence of discomfort or a PVR of 50–300 ml. Therefore, the heterogeneity among these studies was large. Test for between-studies heterogeneity of 15 studies yielded an *I*^2^ of 70%. The pooled cure rate was 93.24% in the acupuncture group and 72.75% in the neostigmine group; the RR (and 95% CI) was 1.28 (1.16, 1.42) when these two groups were compared in the random-effect model, suggesting that acupuncture had a higher effectiveness rate than intramuscular injection of neostigmine in the treatment of postpartum urinary retention. The results of meta-analysis are shown in Figure 4.

#### 3.4.4. Adverse Events

Seven of the 15 studies reported adverse events. Among them 6 studies [11, 12, 14, 19, 21, 24] reported that there was no adverse event. In the remaining one study, one patient from the neostigmine group suffered from full-body tremor 5 minutes after the intramuscular injection of neostigmine, which was spontaneously resolved 10 minutes later; no other abnormality was found except for slightly higher blood pressure and heart rate; in contrast, no adverse event was found in the acupuncture group [15].

### 3.5. Publication Bias

**Figure 5:**
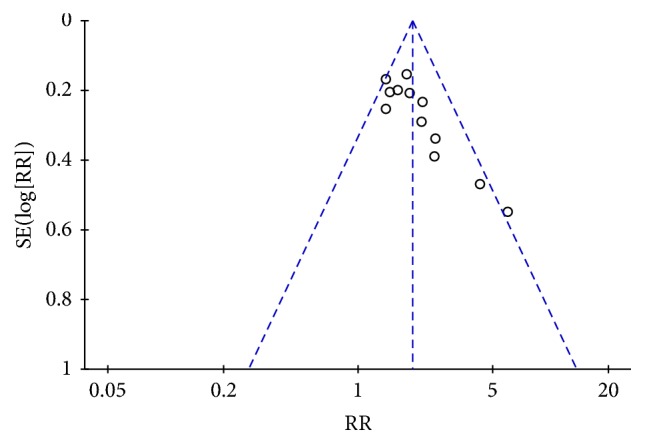
Funnel plot analysis of the cure rate.

Over ten articles described pooled cure rate and effectiveness rate, and their publication biases were analyzed by using funnel plot analysis. The inverted funnel in Figure 5 is asymmetrical between left and right, suggesting that there may be a publication bias.

## 4. Discussion

### 4.1. Principal Findings

Based on our meta-analysis, the cure rate and effectiveness rate were significantly better in the acupuncture alone group than in intramuscular injection of neostigmine alone group (RR and its 95% CI: 1.91 [1.66, 2.19] and 1.28 [1.16, 1.42], respectively). Thus, although intramuscular injection of neostigmine is the most commonly used drug for postpartum urinary retention, its efficacy is suboptimal; instead, acupuncture can achieve better curative effect. Moreover, no adverse event was reported in the acupuncture group, demonstrating acupuncture is safe and reliable. Therefore, acupuncture is more effective and safe in treating postpartum urinary retention.

### 4.2. The Limitations of These Studies

(1) This analysis only included clinical studies comparing acupuncture alone versus intramuscular injection of neostigmine. Studies on other nondrug traditional Chinese therapies (moxibustion therapy) or combined therapies were not included, which was based on the following two considerations. First, moxibustion can produce a bitter taste, which may have certain effects on the newborns who typically are staying with the mothers in the same ward. Second, the combined therapies are more complex than monotherapies. If acupuncture alone can achieve good effectiveness without much cost and time, no other therapy is necessary. (2) The disease severity, definition of “effectiveness rate,” and dosage of intramuscular injection of neostigmine differed among different studies. Therefore, there are certain heterogeneities among these studies. The objective of our current analysis was to evaluate the efficacy of acupuncture with the most commonly used drug. If the acupoints and dosage of drugs must be fully consistent, it would be impossible to determine whether most acupuncture studies are valid or not. (3) In some of the studies included, the specific randomization method was not described, blinding was not adopted, and the specific values of PVR were not reported; therefore, the methodological quality of these studies was average. (4) There was no study that compared acupuncture with blank control or placebo, so we did not know the effect of natural cause of the disease and the efficacy of acupuncture.

### 4.3. Implications for Clinical Practice and Further Research

The results of these studies have demonstrated that, as a safe and simple therapy, acupuncture alone has a better efficacy than intramuscular injection of neostigmine. The main acupoints used in these studies included sanyinjiao (SP6), zhongji (CV3), zusanli (S36), guanyuan (CV4), yinlingquan (SP9), sanjiao (CO17), shuidao (S28), and zhiyin (B67). Since TCM departments have been established in most maternity hospitals and general hospitals in China, it is easy and feasible to apply this treatment.

The included studies were limited by their small sample sizes and relatively low methodological quality. Therefore, prospective randomized controlled trials with larger scale and more rigorous design should be carried out to further validate the clinical efficacy of acupuncture in the treatment of postpartum urinary retention. Future studies should also be focused on the screening of fixed and effective acupoints, so as to benefit more mothers with this condition.

## 5. Conclusion

This review of 15 randomized trials shows that acupuncture is more effective and safe in treating postpartum urinary retention compared with intramuscular injection of neostigmine. However, the beneficial findings are inconclusive due to generally moderate evidence, and further large, rigorous trials are still warranted.

## Figures and Tables

**Table 1:** Features of the included studies.

Study ID	Sample size	Cause	Age (years)	Treatment versus control	Outcome
Bao and Yuan 2010 [11]	50	10 h–5 d after delivery	21–37	Acupuncture group: the needles were punctured into acupoints including guanyuan, zhongji, bilateral zusanli, sanyinjiao, and taixi and retained for 20 min; the needles were punctured into acupoints including shenshu, sanjiao, and weiyang and retained for 10 min. The procedure was performed on a daily basis for 3 days Control group: intramuscular injection of neostigmine 1 mg, qd for 3 days	ER, AE

Chen and Zheng 2012 [12]	65	Inability to urinate after a single catheterization procedure	20–36	Electroacupuncture group: the needles were inserted into acupoints including taiji, qihai, bilateral shuidao, and daju and retained for 30 min (once) Control group: intramuscular injection of neostigmine 1 mg (once)	ER, AE

Chen 2007 [13]	80	Within 3 days after delivery	T: (21–34) C: (22–35)	Electroacupuncture group: the needles were inserted into acupoints including zhongji, qugu, zusanli, and sanyinjiao and retained for 30 min (once or twice) Control group: intramuscular injection of neostigmine 0.25 mg (once)	ER

Gao 2011 [14]	50	Inability to urinate within 8 hours after delivery	N/A	Electroacupuncture group: the needles were inserted into acupoints including hegu, neiguan, sanyinjiao, and yinlingquan and retained for 30 min (once daily for three days) Control group: intramuscular injection of neostigmine 1 mg, qd or bid for 3 days	ER, AE

Hu 2015 [15]	60	1–5 days after delivery	20–35	Acupuncture group: the needles were inserted into acupoints including yinjiao, shimen, guanyuan, zhongji, tianshu, wailing, daju, shuidao, sanyinjiao, and taichong and retained for 30 min (twice daily for 2 days) Control group: intramuscular injection of neostigmine 1 mg (once)	ER, PVR, AE, UT

Kang 2012 [16]	40	4–6 hours after delivery	T: (28.78 ± 2.97) C: (28.00 ± 2.97)	Electroacupuncture group: the needles were inserted into acupoints including zhongwan, tianshu, guanyuan, zhongji, yinlingquan, sanyinjiao, and zusanli and retained for 30 min (once) Control group: intramuscular injection of neostigmine 0.5 mg (once)	ER

Li 2009 [17]	46	5–10 days after delivery	20–31	Acupuncture group: the needles were inserted into acupoints including baihui, zhongji, zusanli, and sanyinjiao and retained for 30 min (qd for 3 days) Control group: intramuscular injection of neostigmine 0.5–1 mg, qd for 3 days	ER

Lu 2007 [18]	84	6–14 hours after delivery	T: (21~39) C: (22~38)	Acupuncture group: the needles were inserted into acupoints including ququan, sanyinjiao, and zusanli and retained for 30 min (twice daily for 3 days) Control group: intramuscular injection of neostigmine 0.5 mg, qm for 3 days	ER

Ning and Zhang 2013 [19]	60	6–8 hours after delivery	T: (31.35 ± 2.54) C: (33.22 ± 2.06)	Acupuncture group: the needles were inserted into acupoints including shuidao, zhongji, guanyuan, sanyinjiao, and zhiyin and retained for 30 min (once daily for 3 days) Control group: intramuscular injection of neostigmine 0.5–1 mg, qd for 3 days	ER

Teng 2017 [20]	66	4–6 hours after delivery	T: (29.68 ± 3.97) C: (28.00 ± 3.32)	Electroacupuncture group: the needles were inserted into acupoints including shuifen, yinlingquan, sanyinjiao, and zusanli and retained for 30 min (once) Control group: intramuscular injection of neostigmine 0.5 mg (once)	ER, AE

Wang 2014 [21]	60	1–5 days after delivery	T: (24.4 ± 2.5) C: (24.6 ± 2.4)	Electroacupuncture group: the needles were inserted into acupoints including zhongwan, xiawan, qihai, guanyuan, zhongji, shuidao, and sanyinjiao and retained for 25 min (once) Control group: intramuscular injection of neostigmine 1 mg (once)	ER

Wu 2004 [22]	90	Inability to urinate within 8 hours after delivery	22–42	Acupuncture group: the needles were inserted into acupoints including zusanli and neiguan and retained for 20 min (once) Control group: intramuscular injection of neostigmine 1 mg (once)	ER, PVR, AE

Wu 2005 [23]	56	1–5 days after delivery	T: (24~46) C: (23~45)	Electroacupuncture group: the needles were inserted into acupoints including zhongji, guanyuan, qihai, zusanli, yinlingquan, and sanyinjiao and retained for 20 min (once daily for 5 days) Control group: intramuscular injection of neostigmine 1 mg, qd for 5 days	ER

You 2003 [24]	68	1–8 days after delivery	T: (22~47) C: (21~48)	Acupuncture group: the needles were inserted into acupoints including shenmen, jiaogan, pizhixia, sanjiao, pangguang, waishengzhiqi, and niaodao and retained for 5–10 min (1–3 times daily) Control group: intramuscular injection of neostigmine 0.5 mg (bid)	ER

Zhao and Yue 2001 [25]	78	6–14 hours after delivery	T: (27.43 ± 4.49) C: (26.00 ± 3.58)	Acupuncture group: the needles were inserted into acupoints including liniao, zhongji, yinlingquan, sanyinjiao, and fuliu and retained for 30 min (once) Control group: intramuscular injection of neostigmine 1 mg (once)	ER, AE

*Note*. ER: effectiveness rate; PVR, postvoiding residual; UT, urination time; AE, adverse event.
